# Plasmonic enhancement of betanin-lawsone co-sensitized solar cells via tailored bimodal size distribution of silver nanoparticles

**DOI:** 10.1038/s41598-020-65236-1

**Published:** 2020-05-19

**Authors:** S. Sreeja, Bala Pesala

**Affiliations:** 1 Academy of Scientific and Innovative Research (AcSIR), 600113 Chennai, India; 2 CSIR - Central Electronics Engineering Research Institute (CSIR-CEERI), CSIR Madras Complex, Taramani, 600113 Chennai, India

**Keywords:** Solar cells, Solar cells

## Abstract

Natural pigment-based photosensitizers are an attractive pathway for realizing low cost and environmentally friendly solar cells. Here, broadband light-harvesting is achieved using two natural pigments, betanin and lawsone, absorbing in the green and blue region of the solar spectrum respectively. The use of bimodal size distribution of AgNPs tailored for each of the pigments to further increase their efficiency is the key feature of this work. This study demonstrates a significant enhancement in current-density, voltage, and efficiency by 20.1%, 5.5%, and 28.6% respectively, in a betanin-lawsone co-sensitized solar cell, via plasmonic enhancement using silver nanoparticles (AgNPs). The optimum sizes of the nanoparticles have been calculated by studying their optical response and electric field profiles using Finite Difference Time Domain (FDTD) simulations, aimed at matching their resonant wavelengths with the absorption bands of the dyes. Simulations show that AgNPs of diameters 20 nm and 60 nm are optimum for enhanced absorption by lawsone and betanin respectively. The FDTD simulations of the plasmonic photoelectrodes demonstrated 30% and 15% enhancement in the power absorption by betanin and lawsone at the LSPR peaks of the 60 nm and 20 nm AgNPs respectively. An optimum overall concentration of 2% (v/v) and a ratio of 4:1 (20 nm:60 nm) of the bimodal distribution of the AgNPs, was determined for incorporation in the photoanodes. An average efficiency of 1.02 ± 0.006% was achieved by the betanin-lawsone co-sensitized solar cell with the bimodal distribution of AgNPs, compared to 0.793 ± 0.006% achieved by the non-plasmonic solar cell of otherwise identical configuration. Electrochemical impedance spectroscopy confirmed that the incorporation of the bimodal distribution of AgNPs in the solar cells also enabled enhanced electron lifetime and reduced recombination compared to the non-plasmonic counterpart, thereby improving the charge transfer. The plasmonic enhancement methodology presented here can be applied to further improve the efficiency of other natural dye-sensitized solar cells.

## Introduction

Since the seminal work in 1991 by O’Regan and Gratzel on Dye-Sensitized Solar Cells (DSSCs)^1^, immense interest has been directed towards their development in the last two decades. Owing to their inexpensive and facile processing requirements, DSSCs are becoming increasingly popular as an emerging photovoltaic technology^2–4^. State-of-the-art DSSCs, which have achieved maximum efficiencies of 11.9%^5^, widely use metal-based dyes as the photosensitizer. Although these dyes are highly efficient sensitizers that effectively capture the entire visible spectrum, they employ rare metals such as ruthenium or osmium which require elaborate synthetic procedures. Moreover, they are expensive and toxic to the environment, making their disposal a problem. Therefore, natural pigments derived from plant sources, which are environment-friendly and inexpensive^6,7^, have come to the forefront as alternative photosensitizers. Several groups^8,9^ have been investigating anthocyanins^10,11^, betalains^12,13^, carotenes^14^, and chlorophylls^15,16^ for their application in DSSCs. To date, average efficiencies achieved for natural DSSCs are ~ 0.4%, with the highest being ~ 1.5 – 2%^8,10–17^. Despite their several advantages, natural dye-sensitized solar cells (NDSSCs) exhibit low efficiencies and stabilities due to insufficient charge separation and limited light-harvesting. Improving the efficiencies of NDSSCs would render them an appealing technological innovation for application in low-light harvesting applications^18^, portable, disposable electronics^19^. Several groups have worked on various aspects of the design of photoanodes and its constituent materials for developing high-efficiency DSSCs^20–24^. Prior studies have demonstrated the co-sensitization of natural pigments for improved light harvesting and better photoelectric conversion efficiencies^25–31^. The present study examines the efficiency enhancement of a betanin-lawsone co-sensitized solar cell by the plasmonic route. The plasmonic route of efficiency enhancement has been explored in various solar cells^32,33^, hydrogen splitting^34^ but not extensively explored for augmenting the efficiency of NDSSCs. The solar cell uses a blend of two natural plant-based dyes: betanin and lawsone, harvesting electromagnetic radiation in the green and blue wavelength range respectively, of the solar spectrum. Betanin is a purplish-red pigment obtained from *Beta vulgaris* (beetroot). It is a glycoside of betacyanin, consisting of a betaine moiety (indole-2-carboxylic acid) N-linked to betalamic acid (pyridine dicarboxylic acid), via an acetyl group^35^, absorbing in light the wavelength range of 450 nm – 600 nm, covering most of the green region. A considerable segment of the incident solar spectrum^36^. The absorption peak of betanin, which is 535 nm in water^12^ and 545 nm in TiO_2_^37^_,_ results from the electron transitions that occur within its conjugated systems. It also has favorable functional groups, i.e. carboxylic groups (-COOH), that facilitate strong anchoring with the TiO_2_ surface through bi-dentate chelation^32^ thereby enhancing the charge transfer efficiencies to the TiO_2_ anode. Lawsone, chosen as the dye complementary to betanin in this study, absorbs both in the UV (280 nm – 400 nm) and visible region (between 400 nm – 550 nm) of the solar spectrum^38^. It is a reddish-orange colored dye (2-hydroxy-1, 4 naphthoquinone), present in the leaves of *Lawsonia inermis* (henna). The absorption of this dye in the visible region is mainly due to the electronic transitions occurring in the quinoid ring. Theoretically, it has been established that there is an appreciable percolation of electron density in lawsone molecules through intermolecular hydrogen bonding^38^. This suggests that lawsone molecules can efficiently transfer electrons upon photo-excitation and therefore are a good potential candidate as a photosensitizer for application in solar cells. The dye in DSSCs plays the vital role of absorbing light to photo-excite electrons into the conduction band of the semiconductor^39^, significantly influencing the performance of the device. An emerging transformational pathway to enhance light harvesting by the dyes is the utilization of the plasmonic effect of metal nanoparticles^33,40–43^.

At specific wavelengths of incident electromagnetic radiation, a cumulative oscillation of electrons is excited within the metal nanoparticles. This phenomenon, known as Surface Plasmon Resonance (SPR) is due to the constructive interference of the incident electromagnetic field with the surface plasmons of the metals^44,45^. Localized Surface Plasmon Resonance (LSPR), exhibited by bounded metals such as metal nanoparticles, results in enhanced extinction of light and also enhances the electromagnetic field around the metal nanoparticle. This is strongly determined by the size, shape or structure and electric permittivity of the metal nanoparticle and the proximal environment^44^, therefore by tailoring these properties appropriately, they can potentially be used for improved light harvesting, carrier generation and enhancement of photoelectric conversion efficiency of DSSCs^46–50^. Noble metals such as gold and silver exhibit plasmon resonances in the visible and infrared region of the electromagnetic spectrum and have been explored by several groups for its application in photovoltaics^33,46,48,51–60^. The plasmonic enhancement of efficiency in a solar cell is ascribed to one or more of the following: (i) far-field scattering of incident light by the metal nanoparticles^44^, (ii) near-field coupling of electromagnetic fields, wherein the metal nanoparticles generate intense electric fields at the surface that are greater than the incident light by several orders of magnitude. These couple with sensitizers that are in the immediate vicinity, resulting in enhanced light absorption^44^, (iii) Plasmon Induced Resonance Energy Transfer (PIRET), wherein the energy from the metal nanoparticles from the LSPR oscillations is relayed to the semiconductor or proximal dye, thereby promoting the generation of electron-hole pairs and separation of charges^61–63^. Persistent efforts are currently underway by several groups to quantify the relative contribution of each mechanism^45,64–67^ in enhancing the efficiency of the device.

Enhancing the light absorption by the dye by harnessing the plasmonic effect of metal nanoparticles is an effective approach for augmenting the photo-electric efficiency of NDSSCs. Silver (Ag) nanoparticles (NPs) that exhibit SPR in the wavelength range of 400 nm − 800 nm^68^ have been chosen for this study due to their comparatively lower cost, easy availability and facile synthesis procedures. The use of tailored bimodal size distribution of AgNPs for the plasmonic enhancement of absorption by both the dyes is the key feature of this work. The optimum dimensions of AgNPs required for their extinction band to match the absorption band of the dyes were determined using Finite Difference Time Domain (FDTD) simulations, aimed at matching their resonant wavelengths with the absorption bands of the dyes. The simulations demonstrated that to enhance light harvesting by betanin and lawsone, a bimodal distribution of AgNPs of diameters ~ 60 nm and ~ 20 nm needs to be incorporated in the photoanode. This will result in two LSPR peaks that coincide with the absorption peaks of betanin (λ_max_ = 535 nm) and lawsone (λ_max_ = 410 nm) in the co-sensitized photoanode, thereby enhancing the light absorption by these dyes. AgNPs of the optimized dimensions were synthesized by the standard citrate reduction methodology and their spectral properties were systematically investigated using confocal microscopy and spectroscopic methods to match the results from the FDTD simulations. To capture the essential physics demonstrating the plasmonic enhancement in the NDSSCs, a simplified layered structure comprising of NPs embedded in the dye on a TiO_2_ substrate was simulated. The AgNPs were incorporated into the photoanodes at optimized total concentrations and the performance of the solar cells was evaluated. The change in the electron lifetime and the internal resistances of the solar cells upon plasmonic enhancement was assessed through electrochemical impedance spectroscopy (EIS) studies of the solar cells. A study on the effect of incorporation of AgNPs on the degradation of dyes and the lifetime of the solar cells has also been carried out.

## Results and discussion

### Design of the solar cell

**Figure 1:**
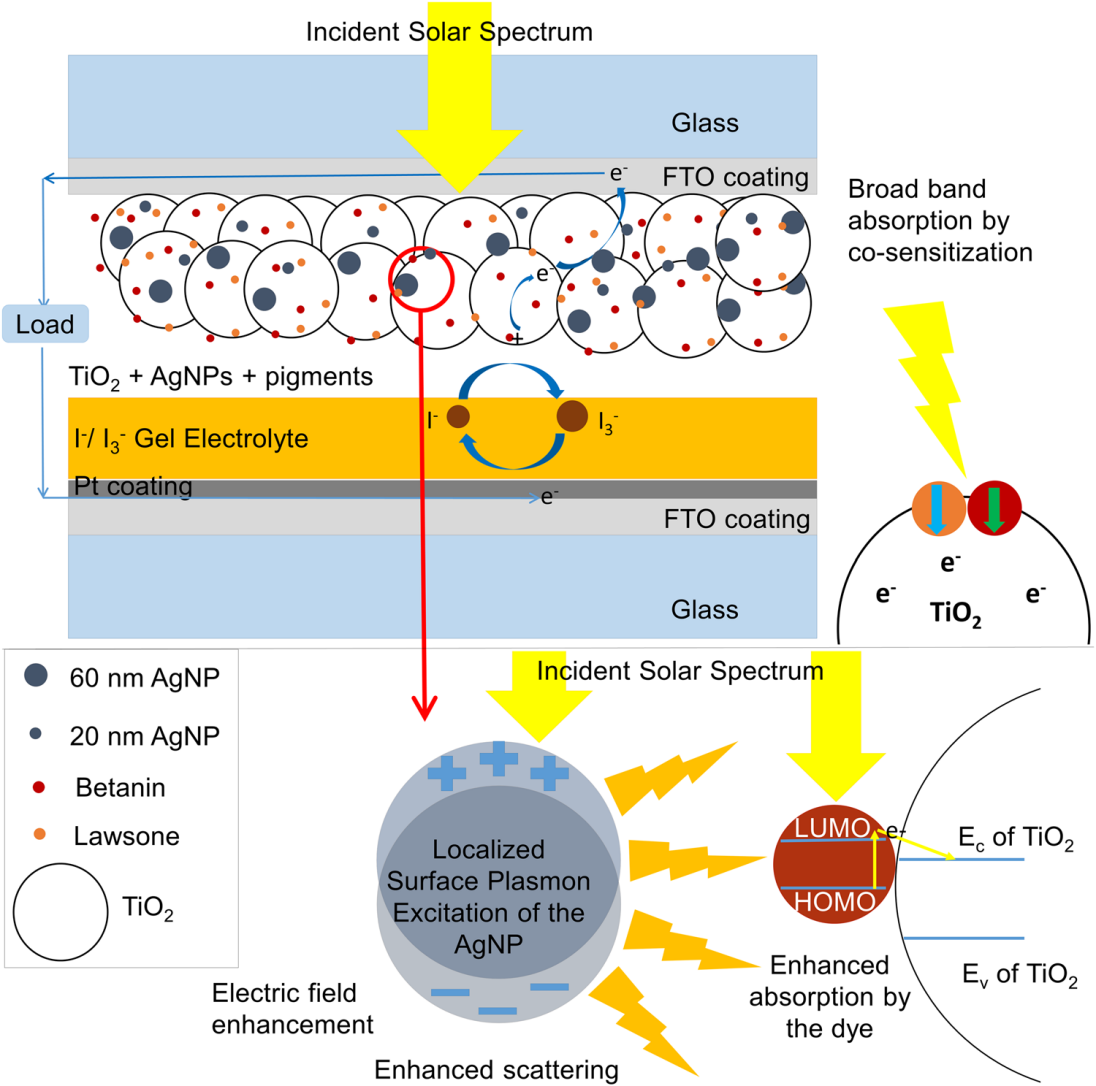
An illustration of the betanin-lawsone sensitized solar cell with the bimodal distribution of 20 nm and 60 nm plasmonic AgNPs incorporated in the photoanode.

Figure 1 is a schematic illustration of the betanin-lawsone co-sensitized solar cell with a bimodal distribution of 20 nm and 60 nm AgNPs incorporated in the photoanode. The structure of the solar cell follows a standard DSSC model^2^. A mesoporous TiO_2_ film which is coated on to the FTO substrate and sensitized with pigments, functions as the photoanode and a Pt-coated FTO substrate as the counter electrode with iodide/tri-iodide redox electrolyte filled in between.

For augmenting the solar cell efficiency by plasmonic means, the AgNPs should have a high extinction cross-section coinciding with the absorption bands of the pigments. The optimal dimensions of AgNPs required for this were determined from FDTD simulations by studying the spectral response and the intensity profiles of their electric fields. AgNPs of the desired sizes were synthesized, characterized, incorporated and tested in various solar configurations.

### Absorption studies of the dyes

**Figure 2:**
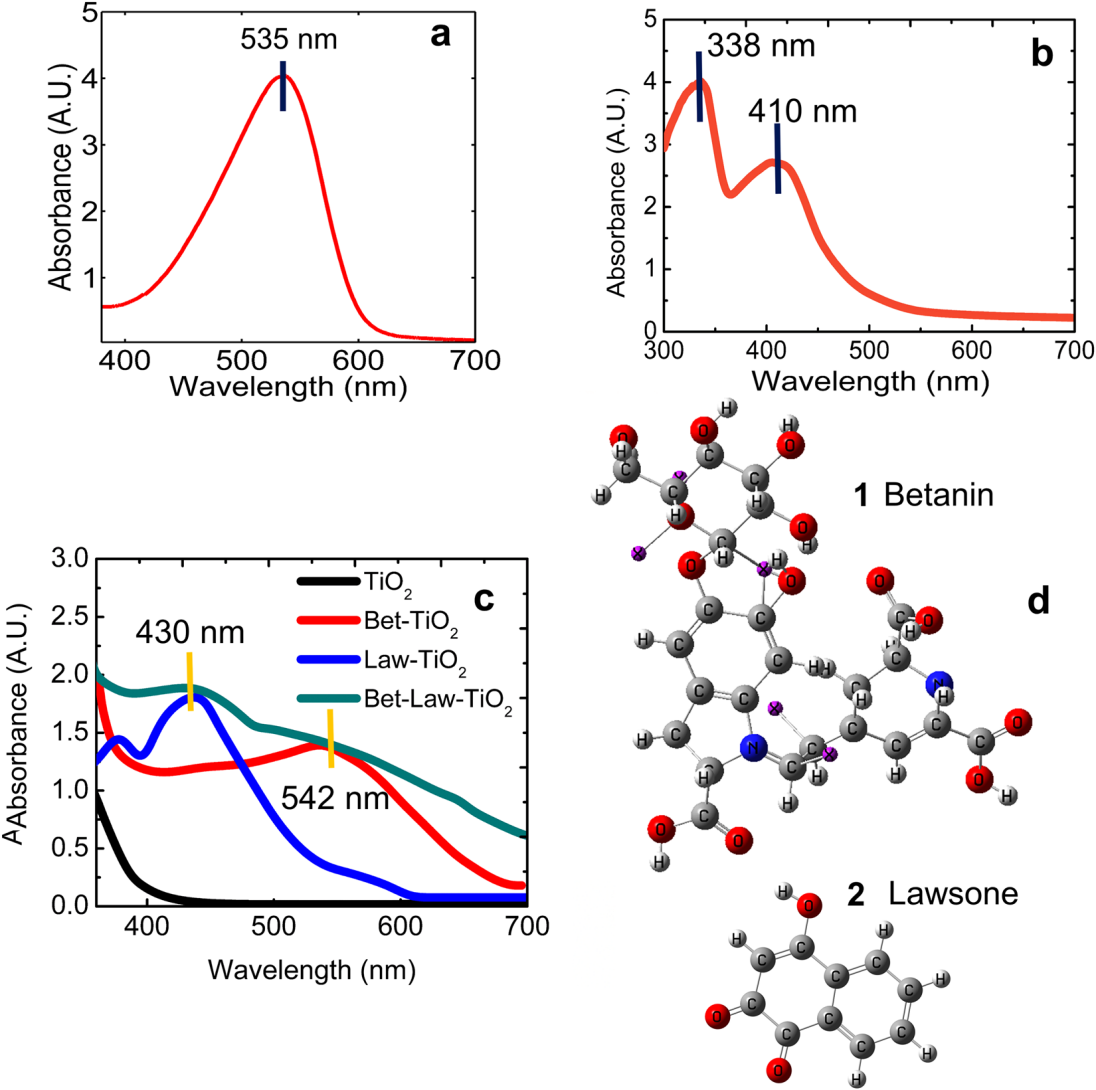
Absorption spectra of (**a**) Betanin dye solution (**b**) Lawsone dye solution and the (**c**) dye-sensitized TiO_2_ photoanodes (**d**) 3D molecular structures of betanin (1) and lawsone (2).

To determine the absorption peaks of interest for the plasmonically augmenting the light absorption by the dyes, their absorption spectra were studied over a wavelength range of 300 nm to 800 nm. Betanin was extracted from beetroot (*Beta vulgaris*) using ethanol as the solvent and its peak absorbance was detected at 535 nm (Fig. 2a), which is typical of betanin^69^. The absorption peak is a result of HOMO → LUMO transitions between the betaine moiety and betalamic acid of the betanin molecule. The pair of nonbonding electrons located on the N-atom of the betaine moiety undergoes delocalization with the electrons located on the conjugated *π* systems. The subsequent n→π* transitions result in the absorption of electromagnetic radiation in the green wavelength range. Lawsone was extracted from *Lawsonia inermis* using acetone as the solvent and its peak absorbance was determined to be at 338 nm and 410 nm (Fig. 2b), characteristic of lawsone, confirmed with the studies reported in the literature^38^. The n→π* transitions in the C=O (and the π → π* transitions in the C=C regions present in the quinoidal ring of lawsone correspond to the HOMO → LUMO transitions which result in the absorption in the UV region i.e. 338 nm. The n→π* transitions are localized around non-bonding electrons of the O-atom and the quinoid ring resulting in the absorption peak in the blue region i.e. 410 nm. As observed in Fig. 2c, when sensitized onto TiO_2_, the absorption peak of betanin shifts to 544 nm. The red-shift of the absorption peaks of the pigment in TiO_2_ as compared to that in water is due to the higher refractive index of TiO_2_^70^. When lawsone is sensitized onto TiO_2,_ its absorption peak shifts to 430 nm (Fig. 2c), due to the change in the surrounding medium^70^ (as observed previously, in the case of betanin as well). Figure 2d shows the 3D molecular structures of (1) betanin and (2) lawsone. Prior studies of the relative NHE (Normal Hydrogen Electrode) levels have confirmed that the LUMO level of both betanin and lawsone, lies above the conduction band of TiO_2_^27,71,72^, necessary for the ideal functioning of the DSSC.

In the present study, both betanin and lawsone are co-sensitized together for broadband absorption. To verify this, both the pigments were coated on TiO_2_ and the absorption spectrum of the co-sensitized photoanode was also characterized, shown in Fig. 2c. As expected, the peak around 430 nm results from absorption by lawsone and the peak at 544 nm results from absorption by betanin (Fig. 2c). The absorption spectrum of the co-sensitized photoanode is broadband overlapping well with the visible portion of the incident solar radiation^36^. For augmenting the efficiency of the solar cell, the absorption peaks of the pigments in the photoanode must match the plasmonic scattering peaks of the incorporated AgNPs. To deduce the optimum size of AgNPs that exhibits LSPR at the wavelengths of 430 nm and 544 nm, FDTD simulations have been performed, discussed in the next section.

### Determination of optimum dimensions of AgNPs through FDTD simulations

#### Study of the spectral response of AgNPs in the betanin-lawsone environment

**Figure 3:**
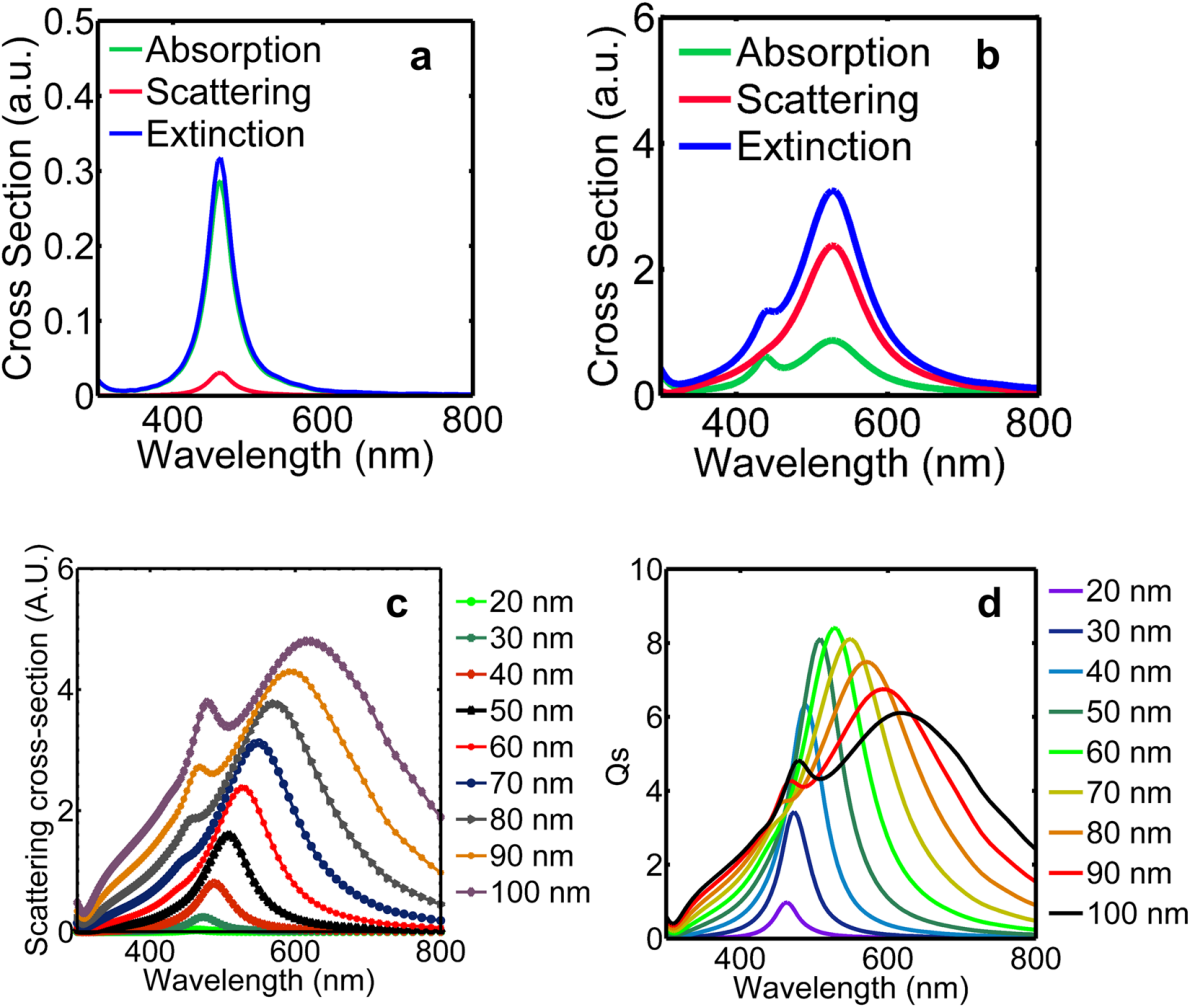
Extinction, absorption and scattering cross sections of AgNPs of diameters (**a**) 20 nm (**b**) 60 nm (**c**) scattering spectra of AgNPs from 20 nm – 100 nm (**d**) normalized scattering cross-section of AgNPs sized 20 nm – 100 nm obtained from the FDTD simulations performed in the betanin-lawsone-TiO_2_ environment.

Lumerical v8.6.2 simulation software was used for performing Finite Difference Time Domain (FDTD) simulations of AgNPs in water, betanin-TiO_2_ environment, lawsone-TiO_2_ environment, and the betanin-lawsone-TiO_2_ environment. The spectral response of the AgNPs of sizes ranging from 20 nm – 100 nm was examined under the visible wavelength range (300 nm – 800 nm). From the simulation results, it could be deduced that the 20 nm AgNPs exhibits strong LSPR peak at 429 nm in the lawsone-TiO_2_ environment and the 60 nm AgNP exhibits a strong LSPR peak at 540 nm in the betanin-TiO_2_ environment^73^. These peak wavelengths from the simulation results coincide well with the experimentally obtained absorption peaks of betanin on TiO_2_, at 542 nm, and that of lawsone on TiO_2_ at 430 nm (discussed in the previous section), and hence AgNPs of 20 nm and 60 nm are suitable for the plasmonic enhancement of lawsone and betanin, respectively. Figure 3a,b show the extinction, absorption and scattering cross-section plots of the 20 nm and 60 nm AgNPs respectively, in the betanin-lawsone-TiO_2_ environment obtained from the simulations. Figure 3c shows the combined graph of the scattering spectra of the AgNPs of sizes 20 nm – 100 nm, in the betanin-lawsone-TiO_2_ environment. It is observed that the scattering cross-section increases with the increasing diameter of the AgNPs and it is higher than the absorption cross-section for sizes larger than 40 nm. This follows the Mie’s theory^44^, which describes the dependence of the absorption cross-section and scattering cross-section with the 3^rd^ power and 6^th^ power of the diameter, respectively. The red-shift of the resonance peaks observed on altering the surrounding medium from water to betanin-lawsone-TiO_2_ which is due to the greater RI of the latter medium^74,75^. For AgNPs of diameters greater than 50 nm, a second peak of lower intensity occurs at a lower wavelength region as a result of quadrupole resonance, whose oscillation pattern is different from that of the dipole resonance^76^. Q_s_ (normalized scattering cross-section) i.e. the scattering cross-section to the geometric cross-section of the nano-sphere is described as;

1 $${{\rm{Q}}}_{{\rm{s}}}=\frac{{\sigma }_{s}}{{\rm{\alpha }}},$$

where σ_s_ represents the scattering cross-section and

2 $$\alpha =\frac{{\rm{\pi }}}{4}{d}^{2},$$

where ‘d’ is the diameter of the nanosphere. At resonance, AgNPs exhibit scattering cross-sections much larger than absorption cross-sections demonstrating the benefit of plasmonic enhancements. Q_s_ increases with an increase in diameter till diameter 60 nm, however, it decreases beyond this size with the LSPR peaks becoming less intense and broad. Moreover, AgNPs of larger sizes tend to agglomerate making them unsuitable for this application. Figure 3d, which shows the normalized scattering cross-section with respect to the wavelength of the incident light, demonstrates that maximum scattering occurs for the 60 nm AgNPs and the wavelength of scattering concurs with the absorption maximum of betanin (when sensitized onto TiO_2_). AgNPs of size 20 nm displays peak absorption and scattering at a wavelength coinciding with the absorption maximum of lawsone (when in TiO_2_). The intensity profiles of the electric field exhibited by the 20 nm AgNPs and 60 nm AgNPs were studied over the visible spectrum to verify the enhancement at its plasmon resonant wavelength.

#### *Electric field enhancement by the 20* *nm and 60* *nm AgNPs*

From the scattering plots, it is understood that the AgNP of 20 nm size shows an extinction peak at 430 nm, optimum for enhancing the absorption by lawsone in the photoanode, and AgNPs of 60 nm size shows an extinction peak at 540 nm, optimum for enhancing the absorption by betanin in the photoanode. The electric field profiles around the 20 nm and 60 nm AgNPs are investigated in the betanin-lawsone-TiO_2_ environment at various wavelengths of incident light, ranging from 300 nm to 800 nm.

**Figure 4:**
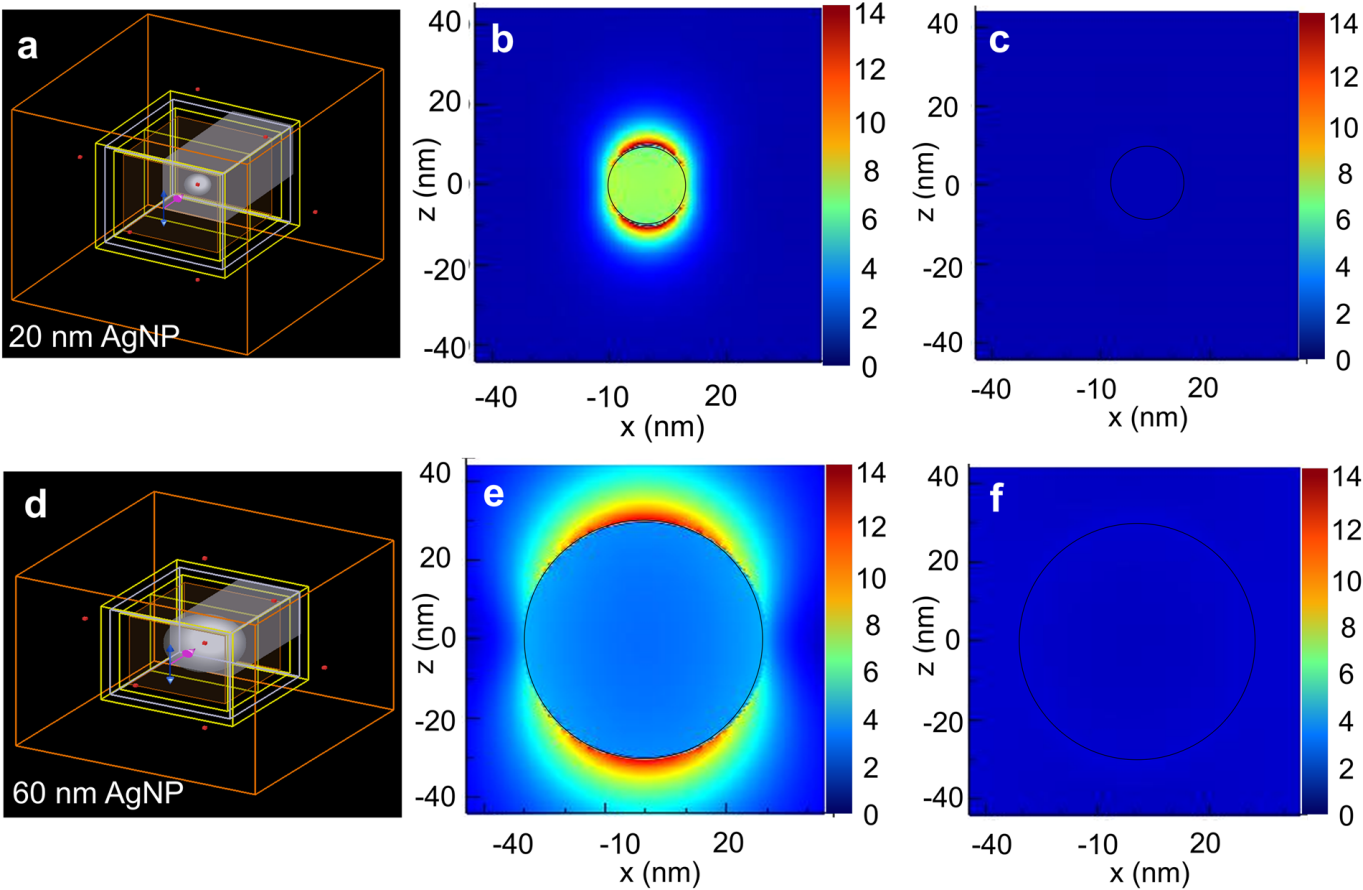
(**a**) FDTD simulation set-up of the 20 nm AgNP in the betanin-lawsone-TiO_2_ environment with a Total Field Scattered Field (TFSF) light source of wavelength range 300 nm – 800 nm. Electric field (magnitude) profiles of the 20 nm AgNP at (**b**) ON resonance (431 nm) and (**c**) at OFF resonance (300 nm). (**d**) FDTD simulation set-up of the 60 nm AgNP in the betanin-lawsone-TiO_2_ environment with a Total Field Scattered Field (TFSF) light source of wavelength range 300 nm – 800 nm. Electric field (magnitude) profiles of the 60 nm AgNP at (**e**) ON resonance (544 nm) and (**f**) at OFF resonance (300 nm).

Figure 4a shows the FDTD layout used for simulation of the 20 nm AgNP in the betanin-lawsone-TiO_2_ environment. The ON resonance wavelength, i.e. the wavelength at which the electron oscillations are maximum and the intensity of electric field the highest, was found to be at 431 nm (Fig. 4b) for the 20 nm AgNP. Figure 4c shows low electric field intensity profiles around the 20 nm AgNP at 300 nm, which is an OFF resonance wavelength. Figure 4d shows the FDTD layout used for simulation of the 60 nm AgNP in the betanin-lawsone-TiO_2_ environment. The ON resonance wavelength for the 60 nm AgNP was found to be at 544 nm (Fig. 4e). The electric field intensity profile of the nanoparticle at an OFF resonant wavelength of 300 nm has been shown in Fig. 4f. At ON resonance, the magnitude of electric field intensity is enhanced than the incident field by a factor of 14, exhibiting an intense plasmonic effect.

In the case of the 20 nm AgNPs, the electric field observed inside the metal nanoparticle is because the size of the particle is of the order of skin depth of silver at this wavelength of incident light^44^. It may also be observed that the dipole observed around the 20 nm AgNP is less distinct. This may be explained by the following: The applied field induces a dipole moment;

3 $${\rm{p}}={\varepsilon }_{{\rm{m}}}\alpha {{\rm{E}}}_{0,}$$

where ε_m_ is the complex permittivity of the metal, E_0_ is the amplitude of the electric field and α is the polarizability, which the measure of how easily charge within a particle may be displaced on the application of electric field^44,77–79^. The polarizability of a particle is proportional to the 3^rd^ power of its diameter^77–79^. Since the 20 nm AgNP is very small, it shows low polarization and low dipole moment. Also, they are highly absorbing and have low scattered fields, as explained by Mie’s theory^44^.

The ON resonance wavelengths of both 20 nm and 60 nm AgNPs are close to the absorption peaks of lawsone and betanin, respectively. These studies demonstrate that AgNPs of diameters 20 nm and 60 nm show enhanced absorption, scattering at the desired wavelength ranges and can be used to enhance the light-harvesting by lawsone and betanin, respectively. AgNPs of the optimized sizes of 20 nm and 60 nm were synthesized in-house and characterized before testing in the solar cells.

### Experimental studies of the synthesized AgNPs

**Figure 5:**
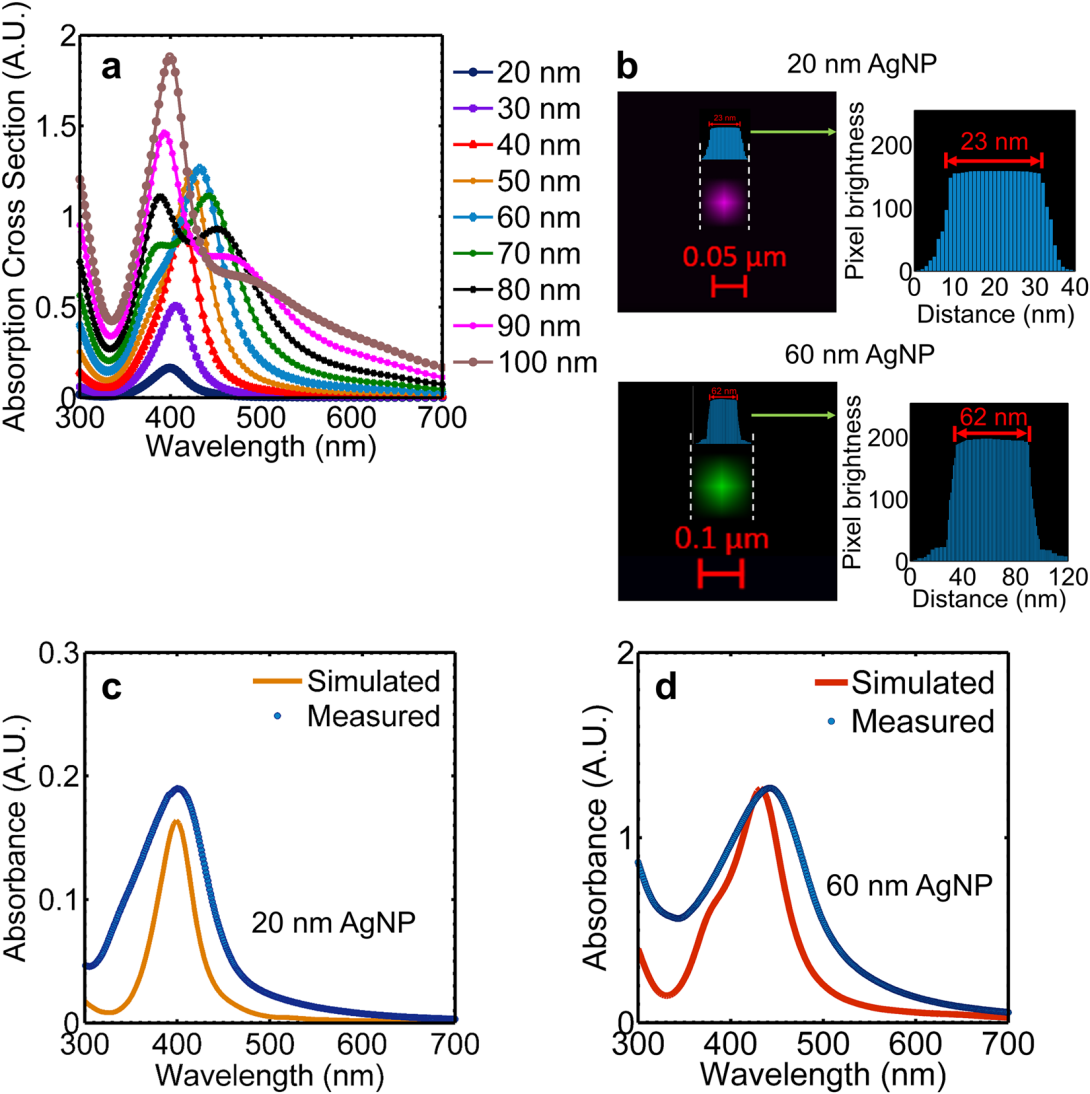
(**a**) Absorption spectra of AgNPs in water for diameters ranging from 20 nm to 100 nm obtained from FDTD simulations^73^. (**b**) Confocal micrographs of the 20 nm and 60 nm AgNPs. Comparison of simulated and measured absorption: (**c**) AgNPs of 20 nm diameter and (**d**) AgNPs of 60 nm diameter showing concurrence with the absorption maxima obtained experimentally.

The absorption spectrum of the colloidal suspensions of the synthesized 20 nm and 60 nm AgNPs were measured across a 300 nm – 800 nm wavelength range and compared with the data determined from the simulations. Figure 5a shows the combined absorption plots of AgNPs in water for diameters ranging from 20 nm – 100 nm derived from the FDTD simulations^73^. This matches experimental data available in the literature^80–82^, hence validating the simulation set-up used in this study. To verify the size and optical properties of the synthesized AgNPs, they were observed using confocal microscopy and their measured absorption curves were matched against the simulated curves.

The synthesized AgNPs were studied using Confocal Laser Scanning Microscopy (LSM-880). The system employed a 405 nm laser and a 458 nm laser for studying the 20 nm AgNPs and 60 nm AgNPs, respectively. The confocal laser scanning micrographs of a single 20 nm and 60 nm AgNP are shown in Fig. 5b. From the image, it can be observed that the AgNPs appear slightly bigger than their expected sizes (determined from the absorbance curves). This size difference is due to the scattering by the nanoparticles occupying more pixels in the image than the nanoparticle itself^83^. This is particularly helpful in the detection of nanoparticles of extremely small sizes such as the 20 nm AgNPs. Image analysis to determine the nanoparticle size was performed using ImageJ/Fiji^84^ using the thresholding methodology^83,85,86^ of the pixel brightness across the diameter of the scattered cross-section observed in the image. Since the array of pixels image a single nanoparticle, counting of the pixels and scaling it with respect to the scale bar of the image would provide an accurate estimation of the total diameter of the observed scattering cross-section of the nanoparticle^83^. The histogram of the pixel brightness across the selected cross-section showed a sudden drop in brightness close to the edges of the cross-section (Fig. 5b). The section of the particle up to which the pixel brightness remains relatively constant is the approximate diameter of the nanoparticle (this excludes the pixels showing scattered light around the nanoparticle). Three AgNPs from the 20 nm and 60 nm sample sets showed sizes of 21.7 ± 2.62 nm and 60.7 ± 1.89 nm respectively. For example, Fig. 5b shows that the measured sizes of one of the nanoparticles from each sample set to be 23 nm and 62 nm respectively.

Figures 5c,d show the measured absorption spectra of the colloidal suspension of the synthesized 20 nm and 60 nm AgNPs (in water), showing absorption maxima at 408 nm and 445 nm, respectively. By matching the experimental and simulated absorption spectra, that the sizes of the prepared AgNPs were deduced to be 20 nm and 60 nm, which is also verified by the highly controlled methodology used to synthesize them^80^. It is observed that although the absorption peaks from the theoretical and experimental results show a good match, the curves do not entirely coincide. This deviation could be because, under experimental conditions, an additional influence of multiple neighboring AgNPs in the chosen environment is expected to have on each other whereas in the simulations only a single AgNP in the chosen environment has been considered. Besides, the simulations use Ag material parameters for the bulk model, though in the nano-scale, the material specifications for AgNPs prepared via solution-processed methods, may slightly differ^87^. The capping agent used for the synthesis could cause result in the broadening of the peaks^88^. Despite adequate precautions followed to guarantee the creation of extremely monodisperse AgNPs according to the prescribed methods^80^, AgNPs of slightly varied sizes may be formed, which could result in the measured absorption curve to be slightly broader compared to the simulated curve.

### Study of the photoelectrodes for plasmonic enhancement

Plasmonic dye-sensitized photoelectrodes were fabricated from the synthesized AgNPs and characterized via absorption and diffuse reflectance studies (see Supplementary Information).

**Figure 6:**
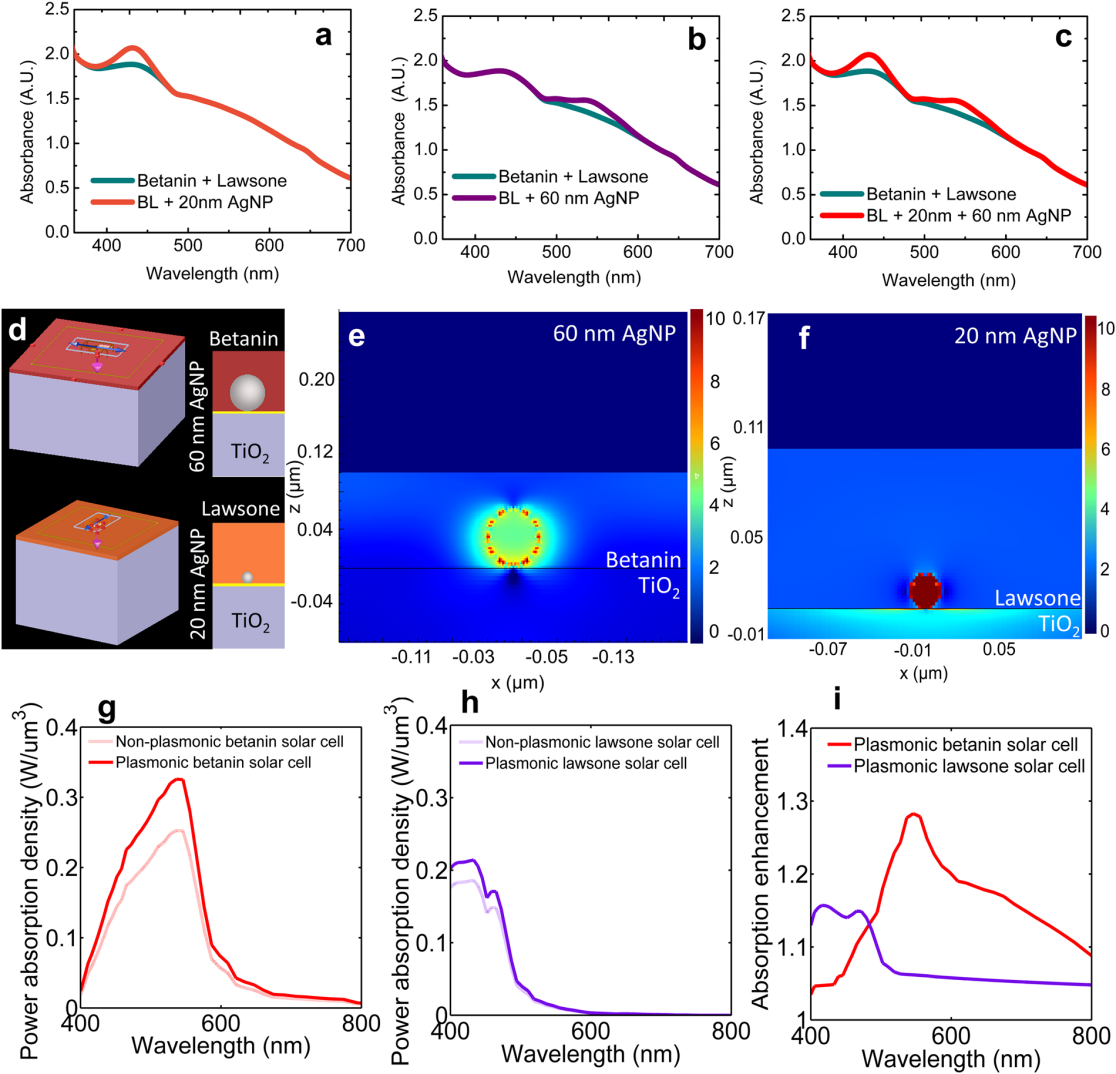
Plasmonic enhancement in absorption spectra of the respective dyes observed (from measurements) in the betanin-lawsone (BL) co-sensitized photoanode on incorporation of monomodal distributions of the (**a**) 20 nm AgNPs (**b**) 60 nm AgNPs and (**c**) bimodal distribution of both 20 nm and 60 nm AgNPs (**d**) FDTD simulation set-up for the plasmonic betanin solar cell and the plasmonic lawsone solar. Mapping of the power absorption density (logarithmic scale) in the (**e**) plasmonic betanin photoelectrode and the (**f)** plasmonic lawsone photoelectrode. Comparison of power absorption density (from simulations) in the non-plasmonic photoelectrodes with that in the (**g**) plasmonic betanin solar cell and the (**h**) plasmonic lawsone solar cell. (**i**) The absorption enhancement (with respect to the non-plasmonic configuration) in the plasmonic betanin photoelectrode and the plasmonic lawsone photoelectrode (from simulations).

Figure 6a–c show the betanin-lawsone co-sensitized photoanodes incorporated with monomodal and bimodal distributions of the 20 nm and 60 nm AgNPs. The plasmonic enhancement resulting in increased absorption by the dyes is observed at their corresponding absorption peaks due to the LSPR effect of the AgNPs.

The plasmonic and non-plasmonic dye-sensitized photo-electrodes were simulated to understand the enhancement in power absorbed in the pigments due to the AgNP incorporation. Figure 6d shows the simulation layout used for the FDTD simulations. The power absorbed within the dye layer was calculated using appropriate script functions in Lumerical^®^ for the betanin and lawsone photoelectrodes with and without the incorporation of AgNPs. Figures 6e,f show the intensity profiles of the optical absorption per unit volume at a selected cross-section of the plasmon-enhanced dye-sensitized photoelectrodes at the SPR wavelengths of the AgNPs incorporated. The absorption density is plotted on a logarithmic scale using the same color scale to facilitate the comparison. Figure 6e shows enhanced absorption into the betanin dye around the 60 nm AgNP at its SPR wavelength (i.e. 544 nm). Likewise, Fig. 6f shows enhanced absorption into the lawsone dye close to the 20 nm AgNP at its SPR wavelengths (i.e. 431 nm). The results indicate that the presence of the 60 nm and 20 nm AgNPs increases absorption by the dyes due to the enhanced scattering and absorption due to the LSPR of the AgNPs in the betanin and lawsone photoelectrodes. It may also be observed that the enhancement in the absorption by the dye due to scattering is higher in the plasmonic betanin photoelectrode compared to the plasmonic lawsone photoelectrode, whereas in the case of the plasmonic lawsone photoelectrode although some enhancement upon absorption by the dye due to scattering by the nanoparticle is seen, the absorption by the 20 nm AgNP itself is higher. This is because the 60 nm AgNP scatters more than it absorbs whereas the 20 nm AgNP absorbs more than it scatters (as mentioned previously).

Figures 6g,h show the power absorption density across the visible wavelength range, by the plasmonic betanin-sensitized photoelectrode and the plasmonic lawsone-sensitized photoelectrode respectively compared against their corresponding non-plasmonic configurations. About 30% absorption enhancement can be observed at the LSPR peak of the plasmonic betanin photoelectrode and about 15% absorption enhancement can be observed at the LSPR peak of the lawsone-TiO_2_ photoelectrode. The absorption enhancement g(λ) for the plasmonic dye-sensitized photoelectrodes calculated with respect to the corresponding non-plasmonic dye-sensitized photoelectrodes (shown in Fig. 6i. g(λ)) compares the efficiency of the plasmonic photoelectrode with a non-plasmonic photoelectrode and is defined as^89,90^;

4 $${\rm{g}}(\lambda )=\frac{QE{(\lambda )}_{plasmonic}}{QE{(\lambda )}_{bare}},$$

where QE (λ) or quantum efficiency is defined as^89,90^;

5 $${\rm{QE}}(\lambda )=\frac{P{(\lambda )}_{abs}}{P{(\lambda )}_{in}},$$

where *P*(*λ*)_*abs*_ and *P*(*λ*)_*in*_ are the power of absorbed light by the photoelectrode and the power of the incident light on the photoelectrode, respectively, at a wavelength λ^89,90^.

The simulation results approximately indicate an enhancement in the dye absorption in the presence of the AgNPs in both cases. It should be noted that the representation of the dye-sensitized photo-electrodes used in the simulations is not the most accurate representation of the mesoporous nature of the TiO_2_ layer and the chemical interaction of the dyes with it. Nevertheless, the demonstrated absorption enhancement will translate to an increase in current density in the plasmonic solar cells, and a combined increase is expected with the bimodal distribution of 20 nm and 60 nm AgNPs in the betanin-lawsone co-sensitized plasmonic solar cell.

### Performance characteristics of the plasmonic solar cells

#### Current density-voltage measurements

**Figure 7:**
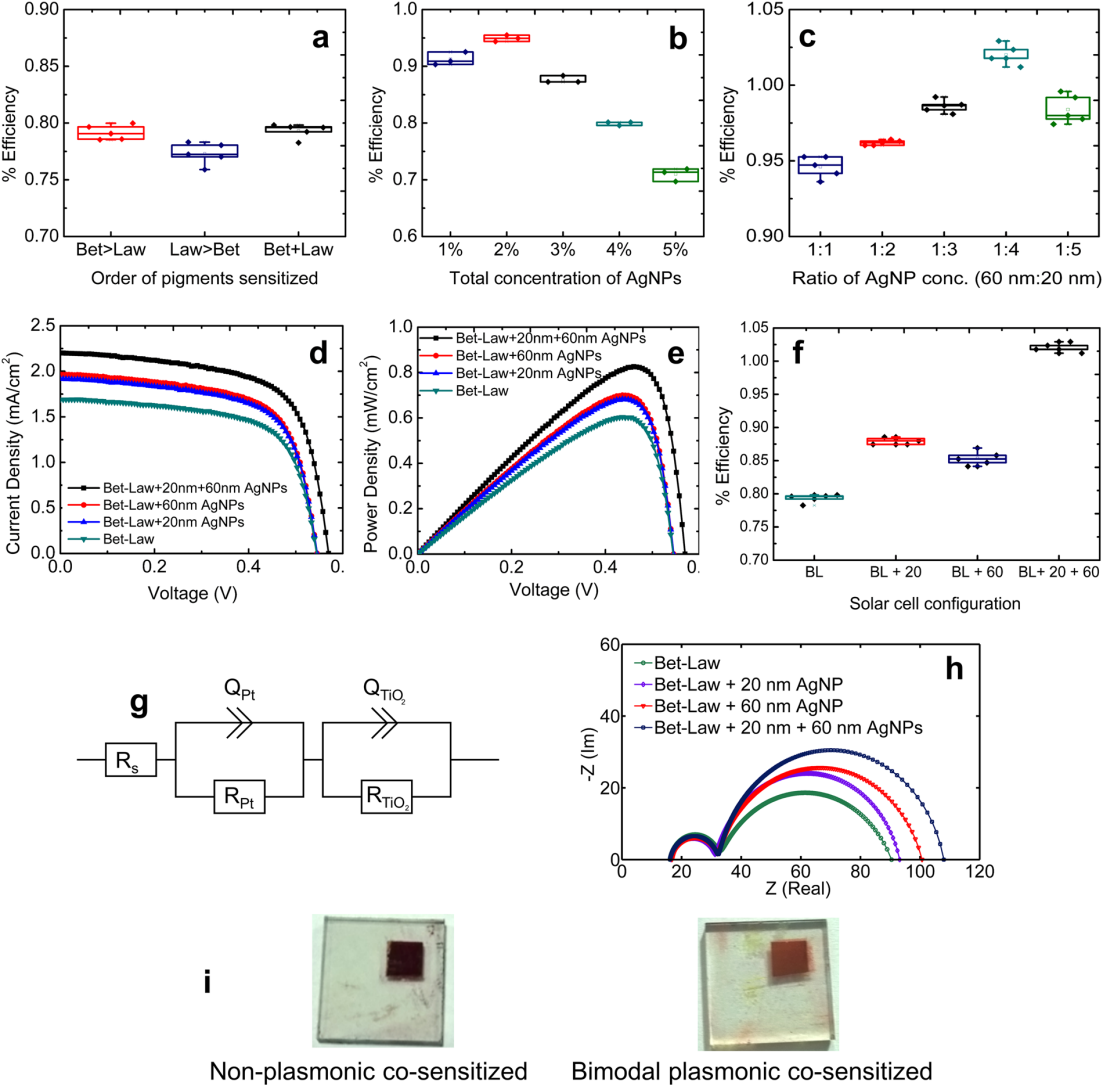
Averaged values of the performance parameters along with the standard deviations are shown for the solar cell configurations tested for the optimization of (**a**) Order of sensitization (**b**) Nanoparticle concentration in betanin-lawsone solar cells containing the 20 nm and 60 nm Ag nanoparticles. (**c**) The ratio of the 20 nm and 60 nm AgNPs in the bimodal distribution incorporated in the betanin-lawsone solar cell. (**d**) Photocurrent density-voltage (J-V) curves and (**e**) Power density-voltage (P-V) characteristic curves of the best performing betanin-lawsone co-sensitized DSSCs, with and without the incorporation of the AgNPs (**f**) Box-plot showing averaged values of the parameters along with the standard deviations for the four solar cell configurations with > 5 samples of each configuration. (**g**) Equivalent circuit model. (**h**) Nyquist plots of the betanin-lawsone solar cell, the betanin-lawsone solar cell with 20 nm AgNPs, the betanin-lawsone solar cell with 60 nm AgNPs and the betanin-lawsone solar cell with 20 nm and 60 nm AgNPs (**i**) Photographs of the non-plasmonic betanin-lawsone co-sensitized solar cell and the bimodal plasmonic betanin-lawsone co-sensitized solar cell.

To test the effect of plasmon enhancement in the photoelectric conversion efficiency, the plasmonic solar cells of various configurations were assembled and their J-V characteristics were evaluated under a standard AM 1.5 G illumination condition of 1000 W.cm^−2^. The performance and the conversion efficiency the solar cells were evaluated and compared by determining the short-circuit current density (J_sc_), open-circuit voltage (V_oc_), fill factor (FF), and conversion efficiency (η), which are determined from the J–V characteristic curves of the cells. First, the optimum order for sensitization of the pigments was determined by coating the pigments in the below-described sequences: (i) betanin-lawsone, (ii) lawsone-betanin (iii) pre-mixed blend of betanin and lawsone at 1:1 ratio (v/v). It was observed that the pre-mixed solution gave better results (average η of 0.793%) in comparison with the solar cells prepared by sensitizing the pigments sequentially (average efficiencies of 0.791% and 0.773% obtained for configuration (i) and (ii) respectively). Each configuration was prepared in quintuplicates and their efficiencies were assessed (Fig. 7a). Next, the optimal concentration of the AgNPs required for the best functioning of the solar cell was determined. The colloidal suspensions of 20 nm and 60 nm AgNPs were mixed with the TiO_2_ paste at (v/v) concentrations varying from 1–5%. It was found that 1% and 4% were the optimum concentrations of 60 nm AgNPs and 20 nm AgNPs to be incorporated with betanin and lawsone, individually. Next, the optimum overall concentration for the incorporation of both sets of nanoparticles was determined by fabricating betanin-lawsone co-sensitized photoanodes with the bimodal distribution of 20 nm and 60 nm AgNPs at a ratio of 1:1. The overall concentration of the nanoparticles was varied from 1% to 5% (v/v) and the efficiencies of the solar cells were evaluated. The best performance was observed with the bimodal distribution of AgNPs at an overall concentration of 2%, clearly evident from Fig. 7b. The photoelectric conversion efficiencies of 3 samples prepared with each concentration are shown in Fig. 7b. Finally, the ratio of the distribution of the 20 nm and 60 nm AgNPs was optimized for the betanin-lawsone co-sensitized solar cell. Here, the overall concentration (v/v) was kept constant at 2% and the ratio of the 60 nm to the 20 nm AgNPs was varied as 1:1, 1:2, 1:3, 1:4, 1:5 (as it was previously determined that the 20 nm nanoparticles were required to be at higher concentration compared to the 60 nm AgNPs). The optimum ratio of the 60 nm AgNPs to the 20 nm AgNPs, was found to be 1:4. The photoelectric conversion efficiencies of 5 samples fabricated with each ratio are shown in Fig. 7c. Figures 7a–c show the distribution of the solar cell efficiencies of quintuplicate samples of each configuration. Following the optimization, the various solar cell configurations (described subsequently) were prepared and tested: (i) betanin-lawsone solar cell, (ii) betanin-lawsone solar cell with 20 nm AgNPs (iii) betanin-lawsone solar cell with 60 nm AgNPs and (iv) betanin-lawsone solar cell with 20 nm and 60 nm AgNPs. (The performance of solar cells individually sensitized with only betanin and lawsone pigments has been described in our earlier publications^27,72^) The J-V and P-V curves of the highest performing sample in each type of solar cell are shown in Fig. 7d,e. Figure 7f illustrates the distribution of the solar cell efficiencies of quintuplicate samples of the various solar cells investigated. Table 1 lists the average photovoltaic performance parameters of these solar cell configurations.

**Table 1:** Average performance characteristics of the optimized configuration of the solar cells.

Solar Cell	Performance Characteristics					
	V_oc_ (V)	J_sc_ (mA.cm^−2^)	V_max_ (V)	J_max_ (mA.cm^−2^)	% Fill Factor (F.F)	% Efficiency (η)
Bet-Law	0.539	1.692	0.436	1.476	69.44	0.793 ± 0.006
Bet-Law + 60 nm AgNPs	0.545	1.960	0.445	1.599	66.48	0.879 ± 0.005
Bet-Law + 20 nm AgNPs	0.543	1.918	0.440	1.552	65.93	0.854 ± 0.009
Bet-Law + 20 nm + 60 nm AgNPs	0.569	2.205	0.460	1.774	66.07	1.020 ± 0.006

The enhancement in J_sc_ in the plasmon-enhanced DSSCs, clearly evident from Fig. 7d can be attributed to the enhanced light absorption of betanin and lawsone due to localized surface plasmons by the bimodal distribution of AgNPs. This demonstrates a significant improvement in current density by ~ 20.1%, a slight increase in voltage by ~ 5.5% and an enhancement in photoelectric conversion efficiency by ~ 28.6% in the bimodal plasmonic betanin-lawsone solar cell. The performance enhancement in the plasmonic solar cells is attributed to the LSPR effect of the AgNPs enabling enhanced absorption by the pigments thereby increasing the current generation. A 5.6% increase in V_oc_ and a 5.5% increase in V_max_ was observed for the bimodal plasmonic solar cell configurations with respect to the non-plasmonic solar cell. The increase of V_oc_ could be ascribed to the more negative level of the quasi-Fermi energy of AgNP TiO_2_ photo-electrode driven by the added AgNPs^91,92^ (the potential difference between the Fermi energy level of the photo-electrode and the redox potential of the electrolyte determines the V_oc_). It has been understood from the literature that better photoelectron production also increases the photovoltage capacity of the solar cells^93,94^. The increase in V_oc_ has been attributed to the photo charging effect, produced by the electron storage in metal nanoparticles, thus driving the Fermi level to more negative potentials^93,94^. Other factors such as greater impedance to recombination, longer electron lifetime and facile charge separation could also drive a negative shift of the Fermi level in turn benefiting V_oc_. However, the exact mechanism contributing to the V_oc_ enhancement needs further investigation. It has been reported in the literature that the incorporation of metal nanoparticles at optimum concentrations in metal-oxide-semiconductor electrodes (such as TiO_2_) can result in an upward shift of its valence band edge, reduce the bandgap energy of the material and enhance the short-circuit current in DSSCs^91^.

#### Electrochemical Impedance Spectroscopy (EIS)

Electrical impedance spectroscopy (EIS) enables an in-depth understanding of the various physical processes that occur in DSSCs. The quintessential Nyquist plots of DSSCs exhibit 2 semicircles of which, the semicircle having the greater real part is correlated with the electron recombination processes occurring at the interface between the photoelectrode and the electrolyte, and the semicircle with the lesser real part is correlated with the electron transfer activities at the interface between Pt counter electrode and the electrolyte^95–97^. The EIS parameters are extracted by fitting the measured Nyquist plots into an equivalent circuit model^96,98^ (shown in Fig. 7g) that mimics the physical processes occurring in a DSSC. Fitting of the measured data into the model for each solar cell configuration was performed model using the Z-Fit function in the E-C Lab^®^ software. χ^2^ (Chi-Square) values of less than ~ 10^−4^ (with an error of less than 1%) were maintained to ensure the best fitting. The impedance Z(f) of the equivalent circuit model used for describing DSSCs is expressed as^96^:

6 $$Z(f)={R}_{s}+\frac{{R}_{Pt}}{{R}_{Pt}{Q}_{Pt}{(j2\pi f)}^{{\alpha }_{1}}+1}+\frac{{R}_{Ti{O}_{2}}}{{R}_{Ti{O}_{2}}{Q}_{Ti{O}_{2}}{(j2\pi f)}^{{\alpha }_{2}}+1},$$

where R_s_ is the series ohmic resistance of the FTO including the transport loss in the electrolyte layer; R_Pt_ and Q_Pt_ are the electron transport resistance and the capacitance respectively, at the Pt electrode/electrolyte interface; R_TiO2_ is the resistance to recombination and Q_TiO2_ is the chemical capacitance of the double layer at the dye-TiO_2_ photoanode/electrolyte interface. Q denotes the Constant Phase Element (CPE), which describes non-ideal capacitive processes that are typically observed in systems such as DSSCs^96^, where the CPE index represented by α deviates from the ideal value i.e 1^98^. The time constant distributions arising from the heterogeneity of the surface roughness and the surface energy at the interfaces result in this type of behavior. α_1_ and α_2_ in (6) are the CPE indices correlated with non-ideal processes occurring interfaces between the Pt-electrode and the and the dye/TiO_2_-photoelectrode and the electrolyte respectively. The extracted EIS parameters are summarized in Table 2. Figure 7h shows the comparison of the Nyquist plots of the 4 configurations of solar cells. Figure 7i shows the bimodal plasmonic and non-plasmonic photo-electrodes fabricated.

**Table 2:** Parameters determined from EIS spectra of the fabricated DSSCs.

Solar Cell	Parameters						
	R_s_ (Ω)	R_TiO2_ (Ω)	Q_TiO2_ (mF⋅s^α −1^)	α	% error	χ^2^	τ (ms)
Bet-Law	16.12	57.83	7.437	0.728	<0.54	2.94*10^−5^	313.79
Bet-Law + 20 nm AgNPs	16.44	61.94	7.000	0.840	<0.49	3.07*10^−5^	369.63
Bet-Law + 60 nm AgNPs	16.78	68.62	7.305	0.816	<0.55	2.52*10^−5^	428.98
Bet-Law + 20 nm + 60 nm AgNPs	16.12	75.67	8.660	0.863	<0.63	2.67*10^−5^	612.78

In this study, the photoanode is being modified and hence is of primary interest, therefore, the second arc in the Nyquist plots, which corresponds to the electron transport and recombination process within dye-TiO_2_ photoanode/electrolyte interface is analyzed (moreover, from the observed results the first arc correlated with the Pt-electrode/electrolyte interface does not change significantly in the all the solar cell configurations as the composition of the counter electrode is the same in all). One of the major limiting factors for the performance of DSSCs is the electron-recombination during their transport across the photoelectrode^99^. Previous studies have reported the minimization of recombination processes in DSSCs due to the “electron-sink effect” caused by metal nanoparticles^100^. This phenomenon occurs by the formation of a Schottky barrier at the interface of the metal nanoparticle and the semiconductor (in this case, the Ag/TiO_2_ interface). The Schottky barrier results in the storage of electrons which are subjected to charge-equilibration with the photo-excited semiconductor, thereby driving the Fermi level towards increasingly negative potentials^101^. This results in an electron sink that inhibits recombination processes in the dye-sensitized photoanode^102–104^.

For the ideal performance of a solar cell, high impedance to electron-recombination is a requirement. The lifetime of electrons (τ) in the photoelectrode is proportionate to the impedance to recombination at the dye-TiO_2_ photoanode/electrolyte interface. Higher impedance to recombination and capacitance implies higher electron lifetimes and a reduced possibility of electron-recombination activities at the dye-TiO_2_ photoanode/electrolyte interface^105^. The efficiency of the cell will reduce if the recombination of electrons takes place in the dye-TiO_2_ photoanode/electrolyte interface. Therefore, a higher value of τ is favorable. Table 2 lists the electron lifetimes of the various solar cells determined by the following relationship^96^:

7 $$\tau ={(Q\ast R)}^{1/{\alpha }_{2}}.$$

The betanin-lawsone solar cell with the bimodal distribution of 20 nm and 60 nm AgNPs shows the higher value of recombination resistance (R_TiO2_ = 75.67 Ω) in comparison to that observed in the non-plasmonic solar cell (R_TiO2_ = 57.83 Ω). The higher value in the plasmonic configuration is attributed to the lower charge recombination and improved charge transfer due to the presence of AgNPs. The electron lifetime determined for the plasmonic solar cell is 612.79 ms, which is higher than the lifetime of 313.79 ms calculated for the non-plasmonic solar cell. Lower recombination rate of electrons occurs when their lifetime is longer thereby resulting in a higher current. This can also be observed from the higher photovoltaic conversion efficiency of the plasmonic solar cell with the bimodal distribution of the AgNPs. The Nyquist plots of the solar cell assemblies with only a monomodal distribution of either 20 nm or 60 nm AgNPs, were also studied for comparison. As expected, the R_TiO2_ values and the electron lifetime values were found to be intermediate to those of the above-discussed configurations.

The betanin-lawsone solar cells comprising the bimodal distribution of the 20 nm and 60 nm AgNPs demonstrate the longest electron lifetime of 612.78 ms. The longer electron lifetime exhibited by the plasmonic solar cells implies that by incorporating AgNPs in the photoanode, the electron transfer mechanism at the dye-TiO_2_ photoanode/electrolyte interface has also been improved. This can be attributed to more conducting pathways created by the presence of metal nanoparticles within the TiO_2_ mesoporous structure confirmed by the higher current densities and better solar cell efficiencies observed in the bimodal plasmonic betanin-lawsone solar cell.

A brief study was carried out to understand the effect of AgNPs incorporation on the photocatalytic degradation kinetics of the pigments betanin and lawsone and the lifetime of the solar cells (see Supplementary Information). The results showed that the efficiency decrease that is normally observed in NDSSCs due to PCA of TiO_2_ is further accelerated due to the incorporation of the AgNPs, slightly negating the positive effect of the nanoparticles on efficiency enhancement. The photodegradation of the pigments from the photocatalytic action of AgNP-TiO_2_ is found to be larger compared to that of TiO_2_, due to the accelerated formation of reactive species by TiO_2_ in the presence of moisture and oxygen. These studies emphasize that to effectively harness the plasmonic effect of nanoparticles for efficiency enhancement of natural dyes in solar cells, the lifetime and stability of the NDSSCs need to be significantly improved. Hence, it is necessary to develop and optimize robust no-heat sealing techniques that suit the fabrication process of NDSSCs. Most of the presently used components in DSSCs are designed for synthetic dyes and hence are not optimal for their use in NDSSCs. The breakthrough for further augmenting the performance and efficiencies of NDSSCs would be to develop alternative materials to render them more compatible with natural dyes. The photocatalytic-degradation of natural dyes can be controlled by using adequate stabilizers and by adopting effective encapsulation techniques similar to that being used in OLED fabrication, which prevent permeation of oxygen or water molecules (such as glass-lid encapsulation with desiccants, Barix multilayer encapsulation using low-temperature deposition methods, photocurable silica nanoparticle embedded nanocomposites for encapsulation^106,107^).

The results of these studies are promising enough to drive future research on improving the stability of such solar cells and further enhancing their efficiency using stacked/mixed configurations involving multiple dyes and nanoparticles tailored to enhance the absorption of each dye. The optimization framework for the plasmonic enhancement of the sensitizers described here can be easily adapted for augmenting the efficiencies of several reported DSSCs.

## Methods

### FDTD simulations of the silver nanoparticle

The optimal sizes of AgNPs for the desired LSPR effect were deduced using Finite Difference Time Domain (FDTD) simulations, performed on Lumerical^®^ v8.6.2 software, (Lumerical Solutions, Inc.)^108^. This a computational tool that employs the discretization of Maxwell’s equations in a three-dimensional space grid to simulate the interaction of electromagnetic waves with materials based on Yee’s algorithm^109^. Ag parameters from Palik material database^110^ were used to perform the 3D simulations. The surrounding environment was set as water, betanin-TiO_2_, lawsone-TiO_2,_ and betanin-lawsone-TiO_2_ as required for each simulation. For simulating the water environment, the refractive index (RI) was set as 1.33^111^, for the betanin-TiO_2_ environment an average refractive of 1.76 (average RI of anatase TiO_2_ and betanin), and for the lawsone-TiO_2_ environment, an average refractive index of 1.76 (average RI of anatase TiO_2_ and lawsone) was set. Likewise, for the betanin-lawsone-TiO_2_ environment, an average refractive index (RI) of 1.74 (average RI of anatase TiO_2_, betanin, and lawsone) was set. The RI of anatase TiO_2_ (annealed at 400–500 °C) = 1.82^112^, RI of betanin = 1.7^113^ and the RI of lawsone = 1.7^114^). Perfectly Matched Layer (PML) boundary conditions, designed to absorb all the outgoing waves, were applied on all the boundaries of the simulation region. The mesh size of the simulation volume was set at 0.3 nm. A Total Field Scattered Field (TFSF) light source of wavelength range 300 nm to 800 nm was used. By using the TFSF source, the simulation region can be demarcated into two distinct regions – one region with the total field (total of the incident and scattered fields), and the other region with only the scattered field. The incident light was injected along the forward direction of the y-axis at a polarization angle of 0°. The analysis groups “power monitors” were positioned in the scattered field region and the total field region to determine the scattering and absorption cross-sections. To observe the electric field intensity profiles of the AgNPs, “frequency profile monitors” were positioned in the total field region. The extinction, absorption and scattering spectra of AgNPs with sizes ranging from 20 nm – 100 nm (varied in steps of 10 nm) were derived through the simulations to determine the optimum dimensions required for plasmonic scattering to coincide with the absorption maxima of the pigments. Optimal dimensions of 60 nm AgNPs for plasmon enhancement of betanin and 20 nm for the plasmon enhancement of lawsone was determined. To quantify electric field enhancement due to LSPR, the intensity profiles of the electric field around the AgNPs of optimized dimensions were observed under incident light of the wavelength range 300 nm – 800 nm to determine their ON and OFF resonant wavelengths.

### FDTD simulations of the non-plasmonic and plasmonic photoelectrodes

The simulation setup for one unit cell of the structure consists of a TiO_2_ substrate of 2 µm * 2 µm * 2 µm coated uniformly with a 0.1 µm dye layer embedded with the AgNPs, in contact with the TiO_2_ layer (Fig. 6d). The n, k material data for TiO_2_ from Devore et. al. (1951)^115^ was used to describe the substrate and the parameters for the AgNPs were chosen from the Palik database^110^. n, k optical constants for betanin and lawsone were calculated (using the Swanepoole methodology^116^) from the reflectance and transmittance data (available in literature^117,118^) and was used to describe the dye layer in the betanin and lawsone solar cells. PML boundary conditions were applied as boundary conditions on all sides to absorb the transmitted and reflected fields and a mesh size of 0.25 nm was set. A plane wave light source was used with a wavelength range of 380 nm to 780 nm. To calculate the power absorbed/scattered into the dye, two power monitors were placed one on top of the dye, and the other at a distance of 0.01 µm above the dye-TiO_2_ interface. The “power absorbed” analysis group was used to determine the power absorption density (W.µm^−3^). The absorption enhancement was calculated using appropriate script functions for the simulation setups with and without the bimodal distribution of AgNPs. Configurations of photoelectrodes studied here are: (i) 60 nm AgNPs on betanin-TiO_2_ photoelectrode and (ii) 20 nm AgNPs on lawsone-TiO_2_ photoelectrode.

### Synthesis of silver nanoparticles

AgNPs of optimized dimensions were prepared using the citrate reduction method optimized by Bastus. *et al*.^80^. The procedure uses silver nitrate and trisodium citrate as the precursors. The chemical reaction of the synthesis can be expressed as follows: 4Ag^+^ + C_6_H_5_O_7_Na_3_ + 2H_2_O → 4Ag° + C_6_H_5_O_7_H_3_ + 3Na^+^ + H^+^ + O_2_↑^119^. 50 mL of an aqueous solution (de-ionized (DI) water was used to prepare the aqueous solutions) with 5 mM sodium citrate and 0.1 mM tannic acid, which was prepared in a three-necked round-bottomed flask and heated to 110 °C in a rota-mantle for 15 min with continuous stirring. A reflux was used to run cooling water and prevent the evaporation of the solvent. To prepare Ag seeds of the appropriate size required for the synthesis of the 20 nm and 60 nm AgNPs, the tannic acid of 0.1 mM concentration was used^80^. 625 μL of AgNO_3_ (20 mM) was fed into the solution after it began boiling, turning the solution yellow. Following this, the seed solution was diluted by removing 10 mL of sample and adding 8.5 mL of DI water. Subsequently, the temperature of the solution was brought down to 90 °C and 250 μL of sodium citrate (25 mM), 750 μL of tannic acid (2.5 mM), and 625 μL of AgNO_3_ (20 mM) were sequentially dropped using a micropipette with a time delay ~ 1 min. This methodology optimized by Bastus *et al.*^80^ results in monodisperse AgNPs with < 10% standard deviation in their size distributions^80^. The repetition 3 times and 9 times respectively, at intervals of 30 min between each step results in a step-by-step pre-calculated increase in the diameter of the AgNPs^80^ forming AgNPs of the desired sizes: 20 nm and 60 nm. The optical properties of the colloidal solution of the synthesized 20 nm and 60 nm AgNPs were characterized using spectroscopic methods and later incorporated into the photo-anodes of the solar cells.

### Preparation of the solar cell

Fluorine-doped tin oxide (FTO) substrates (15 mm × 15 mm × 2 mm; resistivity <10 Ω.cm^−1^ and transmittance >83%, from Solaronix (Solaronix^®^, Switzerland) were thoroughly cleaned and the TiO_2_ paste was prepared according to methods optimized by Ito *et al*.^120^. The 20 nm and 60 nm AgNPs were homogeneously mixed with the TiO_2_ paste at optimized concentrations through ultra-sonication. The photoanodes for the four different solar cell configurations were prepared by dispensing the homogeneous suspension of the TiO_2_ paste mixed with 20 nm and 60 nm AgNP suspensions at appropriate concentrations on to the FTO substrates by the doctor blading technique and subsequently annealed at 450 °C for 1 h^120^. The concentration of nanoparticles and their ratios were optimized based on solar cell performances and has been described in the section *Performance characteristics of the plasmonic solar cells*. Before the TiO_2_ coating, the prepared 20 nm and 60 nm AgNP suspensions of optimized dimensions were spin-coated for 5 s at a rotational speed of ~ 2000 rpm upon the FTO substrates to enable better light harvesting. For the control solar cell, the photoanode was prepared by coating pristine TiO_2_ (without AgNPs) and annealed simultaneously at identical conditions. The thickness of the coating was verified to be an optimal 20–25 µm^120^ observed via the Z-stacking method of imaging using an optical microscope (Carl Zeiss SteREO Discovery V20. Betanin and lawsone pigments were extracted and purified from *Beta vulgaris* slices and *Lawsonia inermis* leaves respectively, by following procedures described in the literature^12,121^. The factors such as solvent, temperature, time and pH were optimized^72^. The photo-anodes were left immersed in the purified pigment extracts for 24 h in the dark for proper adsorption, following which they were gently rinsed with ethanol to remove debris and dried. For optimum performance, pre-and-post TiCl_4_ treatment of the photo-anodes was done to ensure the best efficiencies of the solar cells (according to optimized methods described in the literature^12,120^). Pt-coated FTO substrates, (Solaronix^®^) were used as the counter electrodes. A solution of LiI/I_2_ (1.0 M/0.1 M), in NMP (N-methyl-2-pyrrolidone), was mixed with 6% (w/w) PVDF (polyvinylidene fluoride) to make the gel-electrolyte^122^, which was used in all the solar cells. A spacer with a thickness of 35 µm was attached around the coated portion of the photo-electrodes. This will facilitate the electrolyte to fill up to the optimum thickness (35 µm) required for the electrolyte and also will protect the TiO_2_-coating-free portion of the FTO substrate from short-circuiting on contacting the counter electrode. The Pt-FTO counter electrode is assembled over this (Pt coating positioned to face the inside of the assembly) to build a standard DSSC sandwiched–configuration. Subsequently, the electrolyte is injected into the solar cell assembly via a 0.5 mm diameter hole drilled in the counter electrode and sealed. A photo-active area of 0.25 cm^2^ was defined using a black mask for all the prepared solar cells. The samples were prepared in quintuplicates to confirm the repeatability and reproducibility of the performance realized. The NDSSC fabrication described above was performed at room temperature and ambient laboratory conditions.

### Characterization and measurement

A solar simulator (Newport Corporation, Oriel^®^ Class AAA) and a Keithley^®^ 2440 5 A (Keithley, Inc.) source-meter were employed to determine the current density-voltage characteristics of the solar cells. The performance characteristics of the solar cells were evaluated under 1 sun illumination (AM 1.5 G standard test conditions at 1000 W.cm^−2^). A UV-Vis spectrophotometer (Shimadzu^®^ UV-2400 PC Series) was employed to study the absorption spectra of the liquid and solid samples and the diffuse reflectance of the solid samples across a wavelength range of 300 nm – 800 nm at a sampling interval of 0.5 nm and the slit width set at 5 nm. Confocal laser scanning microscopy (LSM-880, Axio observer; Carl Zeiss^®^, Germany) was employed to observe the plasmonic scattering/fluorescence by the nanoparticles at their resonant wavelengths. These have a high degree of magnification and resolution. The system employed a 405 nm laser and a 458 nm laser for studying the 20 nm AgNPs and 60 nm AgNPs, respectively. A drop of the colloidal nanoparticle suspensions was placed on a glass slide, covered with a cover-slip, and the edges were sealed. The scattering/fluorescence signals of the fixed samples were observed using the Plan-Apochromat 63×/1.4 Oil DIC M27 objective. An electrochemical workstation (Bio-Logic^®^ VMP3B-20) was employed to perform electrochemical impedance spectroscopy studies of the solar cells. A constant DC voltage bias set at 0.5 V (~ V_oc_ of the DSSC) was applied to the solar cells and their impedance parameters were recorded over a frequency range of 100 kHz–10 mHz. The measured Nyquist plots were fitted into an appropriate equivalent circuit using the Z Fit function in EC-Lab^®^ software and the corresponding EIS parameters such as electron- electron-transport resistance, chemical capacitance and the impedance to electron-hole recombination, were extracted. Multiple measurements were recorded to ensure repeatability and reproducibility and they were performed in ambient laboratory conditions.

## Supplementary information

Supplementary Information.
