# Supplementary material for: Plasmonic enhancement of betanin-lawsone co-sensitized solar cells via tailored bimodal size distribution of silver nanoparticles
Source: Sci Rep. 2020 May 19;10:8240. doi: 10.1038/s41598-020-65236-1 (PMC7237482; doi:10.1038/s41598-020-65236-1)
Supplement: Supplementary file 1 — Supplementary Information. [file 41598_2020_65236_MOESM1_ESM.pdf]

Plasmonic enhancement of betanin-lawsone co-sensitized solar cells via tailored bimodal size distribution of silver nanoparticles

Sreeja S[a] & Bala Pesala[a,b†]
[a] *Academy of Scientific and Innovative Research (AcSIR), Chennai – 600113*
[b] *CSIR - Central Electronics Engineering Research Institute (CSIR-CEERI), CSIR Madras Complex, Taramani, Chennai – 600113*

SUPPLEMENTARY INFORMATION

1. Absorption studies of the plasmonic dye-sensitized photoelectrode

To check the AgNP plasmon aided enhancement in light-harvesting by the dyes in the photoanodes, their absorbance and diffuse reflectance were measured at various stages of preparation of the photoanodes. From our earlier study[1], it was observed that the plasmon absorption peak of the 60 nm AgNPs in the TiO_2 photoanode is at 544 nm which coincides with the absorption peak of betanin in the TiO_2 photoanode at 542 nm. From our earlier study[1], it was observed that the plasmon absorption peak of the 60 nm AgNPs in the TiO_2 photoanode is at 544 nm which coincides with the absorption peak of betanin in the TiO_2 photoanode at 542 nm. From our earlier study[1], it was also observed that the absorption peak obtained experimentally for the 60 nm AgNPs incorporated betanin-TiO_2 photoanodes concurs well with the absorption peak determined for the 60 nm AgNPs simulated in the betanin-TiO_2 environment. The incorporation of AgNPs in the betanin-TiO_2 photoanodes resulted in enhanced absorption by betanin (described in our earlier study[1]), which was also corroborated by the diffuse reflectance studies of the plasmonic and non-plasmonic betanin photoanode[1]. This study has been described in detail in our earlier study involving a different photo-electrode configuration, wherein one of the constituents was betanin[1].

In the present study, a similar trend was also observed in the lawsone photoanode. Supplementary Figure S1a shows the absorption spectrum of the lawsone-sensitized TiO_2 photoanode with and without the incorporation of the 20 nm AgNPs. It is observed that the plasmon absorption peak of 20 nm AgNPs in the photoanode is at 429 nm which coincides with the absorption peak of lawsone in the TiO_2 photoanode at 430 nm. Also, it may be noted that the absorption peak obtained experimentally for the 20 nm AgNPs incorporated photoanodes matches well with the absorption peak obtained for 20 nm AgNPs simulated in the lawsone-TiO_2 environment. As in the first case, the 20 nm Ag incorporated photoanode also shows higher diffuse reflectance than the TiO_2 photoanode (Fig. S1b). However, a distinct peak is not observed in this case, possibly because scattering by smaller sized nanoparticles is inherently lower, as mentioned earlier. On sensitization with lawsone pigment, the scattering by the 20 nm AgNPs incorporated photoanode further decreased as a result of absorption by lawsone. The enhanced absorption by lawsone is a result of enhanced electric field oscillations by the 20 nm AgNPs in the vicinity, whose LSPR peak matches the absorption peak of the dye. It may be noted that in both the cases, the dye-sensitized AgNPs-TiO_2 photoanodes after sensitization with the dyes demonstrate lesser scattering compared to the AgNP-TiO_2 photoanode (and higher than dye-TiO_2 photoanode) implying that the dyes absorb most of the extra scattered light from the AgNPs.

The enhanced absorption by the betanin is mainly ascribed to the enhanced scattering by the LSPR of the AgNPs which results in increased optical path length. The observed field enhancement for a broad spectral range by the AgNPs in TiO_2 is a result of LSPR modes in the arbitrarily dispersed AgNPs within the TiO_2 layer[2,3].

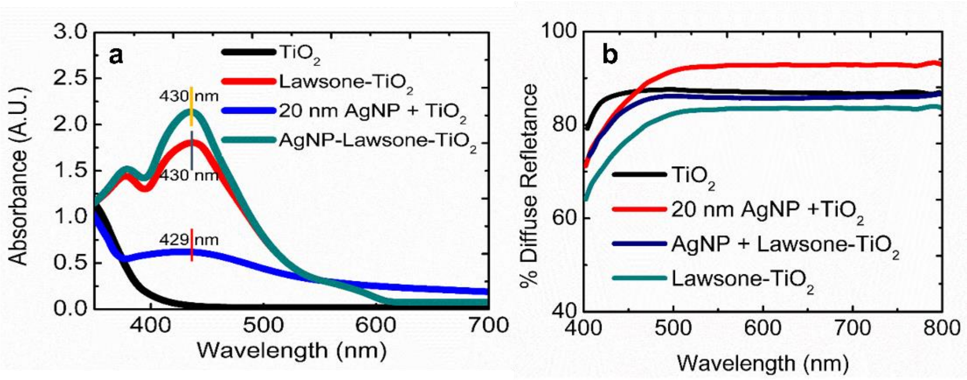

Figure S1 | (a) Absorption spectra of lawsone-TiO_2 photoanodes in the presence and absence of the 20 nm AgNPs (b) Diffuse reflectance spectra of lawsone-TiO_2 photoanodes in the presence and absence of the 20 nm AgNPs

2. Effect of plasmonic AgNPs on the degradation and lifetime of betanin and lawsone solar cells

Natural pigments sensitized onto TiO_2 are generally prone to photocatalytic degradation by TiO_2 due to the presence of moisture and oxygen which seep into it through any gap between the electrodes. Photoexcitation of valence band electrons in TiO_2 to the conduction band occurs upon the absorption of UV light. These high energy excited electrons may interact with water molecules or oxygen and generate free radical ions (mainly reactive OH radicals) which are capable of disintegrating organic matter in the vicinity[4]. As natural dye solar cells are not suitable for sealing by heat-based sealing techniques, moisture and oxygen may permeate, rendering the dyes sensitized onto TiO_2 prone to photocatalytic degradation.

A brief study was carried out to study the effect of AgNPs incorporation on the photocatalytic degradation of the pigments betanin and lawsone. 60 nm and 20 nm AgNPs-TiO_2 coated plates were immersed in betanin and lawsone pigment solutions having an initial concentration of 5 mg.L^{-1} and exposed to 5 h of constant light having an intensity of 1 sun. The change in absorption spectra of betanin and lawsone due to degradation was observed at intervals of 1 h (shown in Fig. S2a and S2b respectively).

The degradation kinetics of betanin and lawsone were also studied and compared against that observed for the pigments in the presence of AgNPs (Fig. S2a and S2b). In both cases, the pigment degradation closely followed first-order kinetics, expressed using the following equation[5]:

$$\log \frac{C_t}{C_0} = -kt, \tag{1}$$

where C_0 is the initial concentration, C_t is the concentration of the dye at a time "t", t is the irradiation time and k is the rate constant. The concentration of the pigments 'C' was estimated using the following relationship[6]:

$$C = \frac{A}{k}, \tag{2}$$

where A is the measured absorbance peak and k is the slope of the standard calibration curves of the pigments (absorbance values plotted against standard concentrations of the pigments). The photocatalytic degradation kinetics of betanin and lawsone which occur normally due to the Photocatalytic Activity (PCA) of TiO_2 can be observed. A decrease in the intensity of absorbance can be observed which implies a degradation of the dye molecules due to the PCA of TiO_2. This could also be observed visually wherein the dye solutions became increasingly decolorized. This is because as molecules are degraded, the degree of delocalization decreases, which means the energy of absorption required for electron transitions increases and therefore λ_{max} decreases[7]. This is observed in the blue-shift of the absorption spectra of both betanin and lawsone towards the shorter wavelength region (seen in Fig. S2a and S2b). Our previous publications[8,9] explored the photocatalytic degradation of betanin and lawsone in the TiO_2 photoanode where 90% degradation was observed for betanin in 4 h and 72% of degradation was observed for lawsone at 5 h. However, the present study demonstrates that the rate of degradation is higher in the presence of AgNPs. In this case, about 98% degradation of betanin is observed within 5 h, and in the case of lawsone, 97% of degradation was observed in 5 h. The kinetic plots of degradation of lawsone and betanin with and without AgNPs were plotted (shown in Fig. S2a and S2b). The adjusted R^2 value of the linear fit for the kinetic plot of betanin degradation is found to be 0.9578 and the “k” values of the degradation kinetic plot of betanin in the presence and absence of AgNPs is 0.006 and 0.003 respectively. The adjusted R^2 value of the linear fit for the kinetic plot of lawsone degradation is found to be 0.9782 and the “k” values of the degradation kinetic plot of lawsone in the presence and absence of AgNPs is 0.005 and 0.001 respectively. Figures S2c and S2d show the percentage decrease in the concentration of lawsone and betanin respectively, in the presence and absence of AgNPs (with respect to time) determined by:

$$\rho = \frac{C_t - C_0}{C_0} * 100, \qquad (3)$$

where ρ represents the percentage decrease in concentration, C_0 represents the initial concentration, t is the irradiation time, C_t denotes the concentration of the dye at a time “t”. This acceleration in photocatalytic degradation of the dyes in the presence of the metal nanoparticles is because they are plasmonic and enhance light scattering within the mesoporous TiO_2 resulting in enhanced photocatalysis of the organic molecules which are in contact with the TiO_2, in presence of moisture and oxygen. Earlier studies have reported that the presence of metal nanoparticles such as Ag, Au, Pt enhances the photocatalytic activity of TiO_2 because they act as electron traps and accelerate the formation of reactive species such as HOO* in the presence of moisture and oxygen, thereby causing faster degradation of the dyes[10,11].

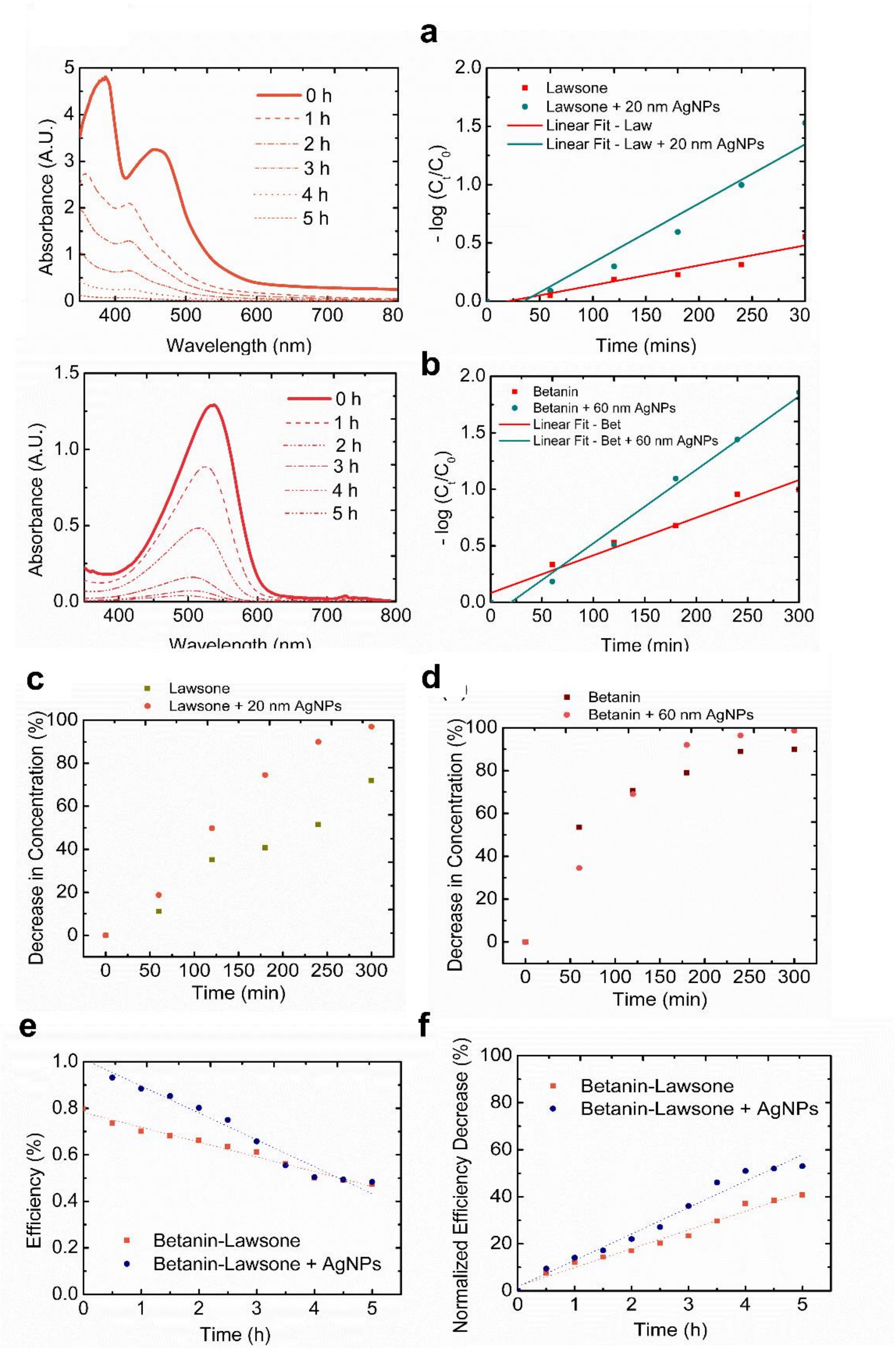

Figure S2 | Effect of plasmonic AgNPs on the degradation of pigments with constant light exposure for 5 h: (a) Change in the absorption spectrum of lawsone, degradation kinetics of the lawsone pigment in the presence of the 20 nm AgNPs (b) Change in the absorption spectrum of betanin, degradation kinetics of the betanin pigment in the presence of the 60 nm AgNPs and (c) the decrease in the concentration of lawsone with respect to time in the presence of the 20 nm AgNPs, (d) the decrease in the concentration with respect to time of betanin in the presence of the 60 nm AgNPs and (f). Change in the efficiency of the two configurations of solar cells under 5 h of constant illumination: (e) absolute efficiency decrease (f) normalized efficiency decrease

To check the effect of nanoparticles on the lifetime of the solar cells, betanin and lawsone solar cells were fabricated were also exposed to constant light having an intensity of 1 sun for 5 h. The change in efficiencies of the solar cells was measured every 30 min. An efficiency decrease of 55% is observed in the case of the betanin-lawsone solar cell incorporated with the bimodal nanoparticles, as compared to a lesser decrease (40% decrease) observed in the non-plasmonic configuration (seen in Fig. S2e and S2f). Though the efficiencies of both configurations after 5 h were almost the same, the bimodal plasmonic configuration started at a much higher efficiency of 1.02% compared to the 0.79% efficiency of the non-plasmonic configuration. This means that the efficiency decrease that is normally observed in natural dye solar cells due to PCA of TiO_2 is further accelerated due to the incorporation of the AgNPs, negating the positive effect of the nanoparticles on efficiency enhancement. The photodegradation of the pigments from the photocatalytic action of AgNP-TiO_2 is found to be larger compared to that of TiO_2, due to the accelerated formation of reactive species by TiO_2 in the presence of moisture and oxygen.

REFERENCES

1. Sreeja, S. & Pesala, B. Efficiency Enhancement of Betanin – Chlorophyll Cosensitized Natural Pigment Solar Cells Using Plasmonic Effect of Silver Nanoparticles. **10**, 124–134 (2020).
2. Lu, H., Ren, X., Sha, W. E. I., Ho, H.-P. & Choy, W. C. H. Broadband near-field enhancement in the macro-periodic and micro-random structure with a hybridized excitation of propagating Bloch-plasmonic and localized surface-plasmonic modes. *Nanoscale* **7**, 16798–16804 (2015).
3. Lu, H. *et al.* Experimental and Theoretical Investigation of Macro-Periodic and Micro-Random Nanostructures with Simultaneously Spatial Translational Symmetry and Long-Range Order Breaking. *Sci. Rep.* **5**, 1–7 (2015).
4. Raja, V., Shiamala, L., Alamelu, K. & Jaffar Ali, B. M. A study on the free radical generation and photocatalytic yield in extended surfaces of visible light active TiO2compounds. *Sol. Energy Mater. Sol. Cells* **152**, 125–132 (2016).
5. Hamad, HA and Sadik, WA and El-Latif, MM Abd and Kashyout, AB and Feteha, M. Photocatalytic parameters and kinetic study for degradation of dichlorophenol-indophenol (DCPIP) dye using highly active mesoporous TiO2 nanoparticles. *J. Environ. Sci.* **43**, 26--39 (2016).
6. B.K. Garg. *Plant Analysis: Comprehensive Methods and Protocols*. (Scientific Publishers (India), 2012).
7. Yan, Z. *et al.* Visible-light degradation of dyes and phenols over mesoporous titania prepared by using anthocyanin from red radish as template. *Int. J. Photoenergy* **2014**, (2014).
8. Sreeja, S. & Pesala, B. Co-sensitization aided efficiency enhancement in betanin-chlorophyll solar cell. *Mater. Renew. Sustain. Energy* **7**, 25 (2018).
9. Sreeja, S. & Pesala, B. Performance enhancement of betanin solar cells co-sensitized with indigo and lawsone: A Comparative Study. *ACS Omega* **4**, 18023–18034 (2019).
10. Begum, T., Gogoi, P. K. & Bora, U. Photocatalytic degradation of crystal violet dye on the surface of Au doped TiO 2 nanoparticles. **24**, 97–101 (2017).
11. Kodom, T. *et al.* Silver Nanoparticles Influence on Photocatalytic Activity of Hybrid Materials Based on TiO 2 P25. **2015**, (2015).
